# Substrate-dependent pore formation in molybdenum disulfide monolayers under ion irradiation

**DOI:** 10.3762/bjnano.17.54

**Published:** 2026-06-12

**Authors:** Yossarian Liebsch, Umair Javed, Lucia Skopinski, Leon Daniel, Franziska Appel, Radia Rahali, Clara Grygiel, Henning Lebius, Carolin Frank, Lars Breuer, Leon Kirsch, Frieder Koch, Jani Kotakoski, Marika Schleberger

**Affiliations:** 1 Fakultät für Physik and CENIDE, Universität Duisburg-Essen, 47057 Duisburg, Germany; 2 University of Vienna, Faculty of Physics, 1090 Vienna, Austria; 3 CIMAP-GANIL, CEA-CNRS-ENSICAEN-UCN, Caen, 14076, France; 4 Materials Physics, Department of Physics and Astronomy, Uppsala University, Uppsala, Sweden; 5 GSI Helmholtzzentrum für Schwerionenforschung, Planckstr. 1, 64291 Darmstadt, Germany

**Keywords:** defects, MoS_2_, nanopores, SiO_2_, scanning transmission electron microscopy (STEM)

## Abstract

Ion irradiation is a versatile tool for nanostructuring surfaces, yet the roles of energy deposition and dissipation at the surface and in ultrathin materials remain poorly understood. In this study, we investigate nanopore formation in monolayer MoS_2_ on different substrates under irradiation of highly charged ions (HCIs) and swift heavy ions (SHIs) – two types of ions that, despite having vastly different kinetic energies, both interact primarily with the electronic system of the target. Using scanning transmission electron microscopy, we quantify pore radii and pore formation efficiencies for suspended MoS_2_, MoS_2_ on SiO_2_, bilayer MoS_2_, and MoS_2_ on gold. Both pore size and pore formation efficiency exhibit a pronounced dependence on the type of substrate. Pores are largest and most frequent in MoS_2_ on SiO_2_, while the gold substrate massively quenches pore formation. The observed pore dimensions under both HCI and SHI irradiation conclusively demonstrate the central role of substrate and interface-dependent electronic dissipation pathways regarding damage under these types of ion irradiation.

## Introduction

Ion beams provide a controllable route to engineer defects in two-dimensional (2D) materials, enabling property tuning from doping to nanopore formation [1–4]. Because kinetic energy, charge state, and mass can be varied over wide ranges, ion irradiation offers a large parameter space for nanostructuring. However, achieving predictive control requires a detailed understanding of ion–solid interaction and post-impact energy dissipation [5]. While ion–bulk interactions are well described across many energy regimes [6–8], the interaction with surfaces and ultrathin targets, particularly for highly charged ions (HCIs) and swift heavy ions (SHIs), remains less complete.

The emergence of 2D materials has intensified the interest in ion–surface interactions [9–13]. Owing to their atomic thickness, 2D materials combine outstanding mechanical properties with promising (opto-)electronic and catalytic functionality [14–18]. Ion irradiation has already proven effective for defect engineering across different energy regimes: Low-energy ions enable implantation with near-atomic precision [19–21], kiloelectronvolt ions primarily create point defects in monolayers [22–24], and HCIs add potential energy that can lead to larger defect complexes [25]. At megaelectronvolt energies, damage is dominated by electronic excitation and can be tuned by the irradiation geometry (e.g., grazing incidence) [26–27].

While fundamental studies of ion irradiation-induced defect formation have largely focused on suspended 2D materials [13,25,28–29], practical applications typically require the 2D material to be supported by a substrate. Direct investigation of ion-irradiated supported 2D materials, however, remains experimentally challenging for several reasons. For example, high-resolution scanning transmission electron microscopy (STEM) generally requires freestanding membranes, while atomic-resolution atomic force microscopy (AFM) and scanning tunneling microscopy (STM) demand exceptionally clean surfaces; also, substrate effects can further complicate interpretation since both topographic and electronic contrast may contain contributions from the substrate as well as from the 2D layer [30–33]. As a result, many studies of supported systems rely on Raman and photoluminescence (PL) spectroscopy, or electrical measurements, which are sensitive to electronic and strain-related changes but do not directly resolve the atomic structure [34–35].

This leaves a gap in our understanding of how ion-induced defect formation differs between suspended and substrate-supported 2D materials. We address this gap by directly measuring substrate effects on defect formation in MoS_2_ under HCI and SHI irradiation. HCIs and SHIs represent two distinct irradiation regimes with vastly different kinetic energies and energy-deposition profiles. Specifically, HCIs release their potential energy predominantly through ultrafast charge exchange and Auger-type processes near the surface [36–37], whereas SHIs deposit energy continuously along their trajectory via electronic stopping *S*_e_. Despite these differences, both ion types couple primarily to the electronic system of the target such that subsequent energy transfer to the lattice is governed by electron–phonon coupling [38–39]. This makes HCI and SHI irradiation a useful pair of probes for testing how substrate-controlled electronic dissipation influences defect formation in 2D materials.

Here, we directly quantify how substrate coupling influences electronically driven defect formation in monolayer MoS_2_. Using STEM, we measure pore radii and pore formation efficiencies after irradiation in several configurations (suspended, supported on SiO_2_, bilayer, and metal-supported samples). We compare HCIs and SHIs as complementary excitation regimes with distinct energy-deposition profiles but a shared dependence on post-impact electronic energy dissipation. Owing to this common feature, we expect that the substrate may influence pore formation in a qualitatively similar way for both ion types.

## Results and Discussion

### Highly charged ions

**Figure 1:**
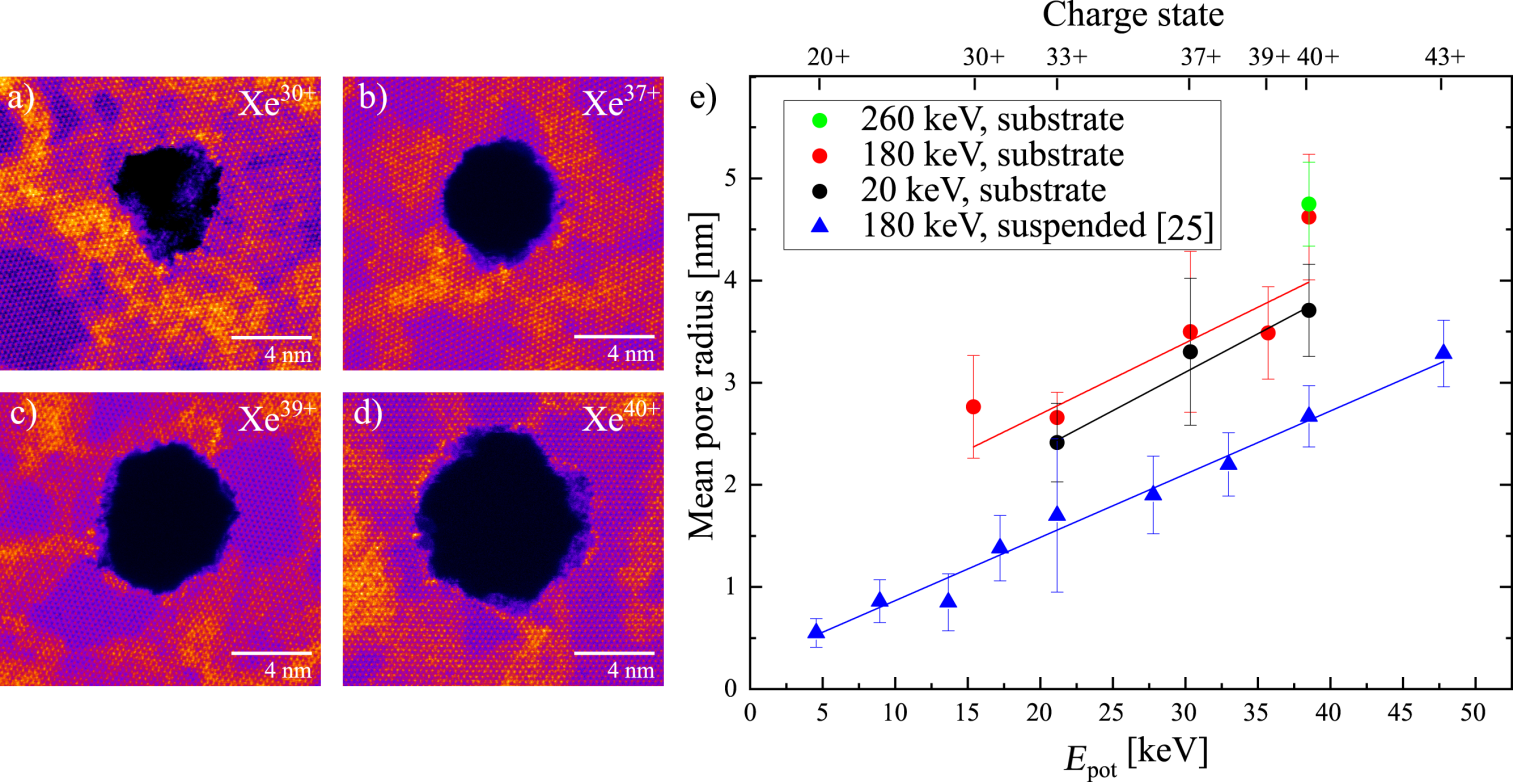
(a–d) False-color STEM ADF images of pores in single-layer MoS_2_ created by Xe ions with different charge states at 180 keV. Orange structures correspond to hydrocarbon contamination. (e) Mean pore radii of monolayer MoS_2_ on SiO_2_/Si substrate irradiated with highly charged Xe ions at different kinetic energies (green, red, black). Pore radii are larger when MoS_2_ is irradiated on SiO_2_ compared to the suspended configuration (blue, data from Kozubek et al. [25]).

In order to assess substrate effects under HCI irradiation, we compare monolayer MoS_2_ on SiO_2_/Si with suspended MoS_2_ reported in one of our previous works [25]. Using the same ion species and kinetic energies as in that work, we vary the charge state from *q* = 30+ up to *q* = 40+, corresponding to potential energies of approximately 15–40 keV. In contrast to earlier AFM studies on supported samples that suggested pronounced topographic changes but could not resolve the defect structure [40–41], our STEM analysis shows that irradiation on SiO_2_ produces well-defined, pore-shaped defects with no obvious long-range lattice distortion of the surrounding material (see Supporting Information File 1, Figure S1), consistent with the pore morphology previously reported for suspended MoS_2_ [25]. Representative pores are shown in STEM annular dark-field (ADF) images in Figure 1a–d.

We quantified pore-size distributions by measuring ≈150 pores. Mean pore radii are summarized in Figure 1e. For both supported and suspended samples, the mean radius increases approximately linearly with the projectile potential energy. Within uncertainty, the slopes are similar for supported and suspended samples (0.06–0.07 nm/keV), indicating comparable scaling with charge state. Pores on SiO_2_ are systematically larger by ≈1 nm across all charge states. In addition, within our experimental resolution, we observe no significant dependence of pore radius on kinetic energy over the investigated range.

This observation is consistent with sputtering experiments of monolayer MoS_2_ carried out by Skopinski et al. [42], who found that the sputtering yield of Mo is significantly more sensitive to changes in the charge state of the HCIs rather than their kinetic energy. These observations suggest that nuclear sputtering contributes only weakly under the present conditions and pore formation is dominated by the deposition and dissipation of the HCIs’ potential energy.

Charge-exchange studies for HCIs on 2D materials carried out by Creutzburg et al. [29] and Niggas et al. [43] found that the majority of the HCIs’ potential energy (≈80–90%) is deposited via Auger processes in the electronic system of the first layer of the target 2D material, independent of the materials electronic properties. In the case of this study, we therefore expect to deliver potential energy in the range of ≈12–36 keV to the monolayer. Linking the charge exchange to pore formation, Grossek et al. [44] introduced a model for nanopore formation in graphene that centers around the target’s charge carrier mobility. They argue that the high electron mobility of graphene leads to a fast charge dissipation in the 2D material, effectively suppressing pore formation. As a consequence, HCI irradiation does not create pores in graphene (with a typical mobility of μ ≥ 60000 cm^2^·V^−1^·s^−1^ for suspended graphene [45]), but is very likely to do so in MoS_2_, which has a significantly lower electron mobility (typically μ ≈ 1–10 cm^2^·V^−1^·s^−1^ for suspended MoS_2_ [46–47]).

Building on the model introduced by Grossek et al., in which charge carrier mobility governs the efficiency of pore formation under HCI irradiation [44], the larger pores observed in MoS_2_ on SiO_2_ can be interpreted in terms of substrate-modified electronic energy dissipation within the MoS_2_ layer. For an insulating substrate, out-of-plane dissipation remains limited. However, substrate-induced disorder and charge trapping are expected to increase carrier scattering, thereby limiting lateral spreading of the non-equilibrium electronic excitation. This is known from several studies [48–49] on charge transport in MoS_2_ that report higher carrier mobility in suspended monolayers than in MoS_2_ on SiO_2_. Reduced mobility can slow lateral charge redistribution, which may increase the spatial localization of the transient excitation and thus raise the local electronic energy density. This in turn may increase the local energy density available for lattice excitation. We note that this interpretation is inferential – the dissipation pathway is not measured directly – but it is consistent with the pore size enhancement observed under both HCI and SHI irradiation (see below Figure 4a), suggesting the effect is not specific to one excitation regime.

The preceding discussion focused on lateral (in-plane) dissipation pathways, treating out-of-plane dissipation as negligible for an insulating substrate. If, however, a semiconducting or metallic substrate is used, this additional dissipation channel could strongly influence pore formation. Multilayer MoS_2_ provides a controlled way to introduce out-of-plane dissipation without changing the substrate material. Although adjacent layers are coupled only weakly via van der Waals forces, a second (or third) MoS_2_ layer can act as an additional reservoir for charge and excitation redistribution relative to an isolated monolayer, offering a simplified analogue of out-of-plane dissipation into a semiconducting environment.

**Figure 2:**
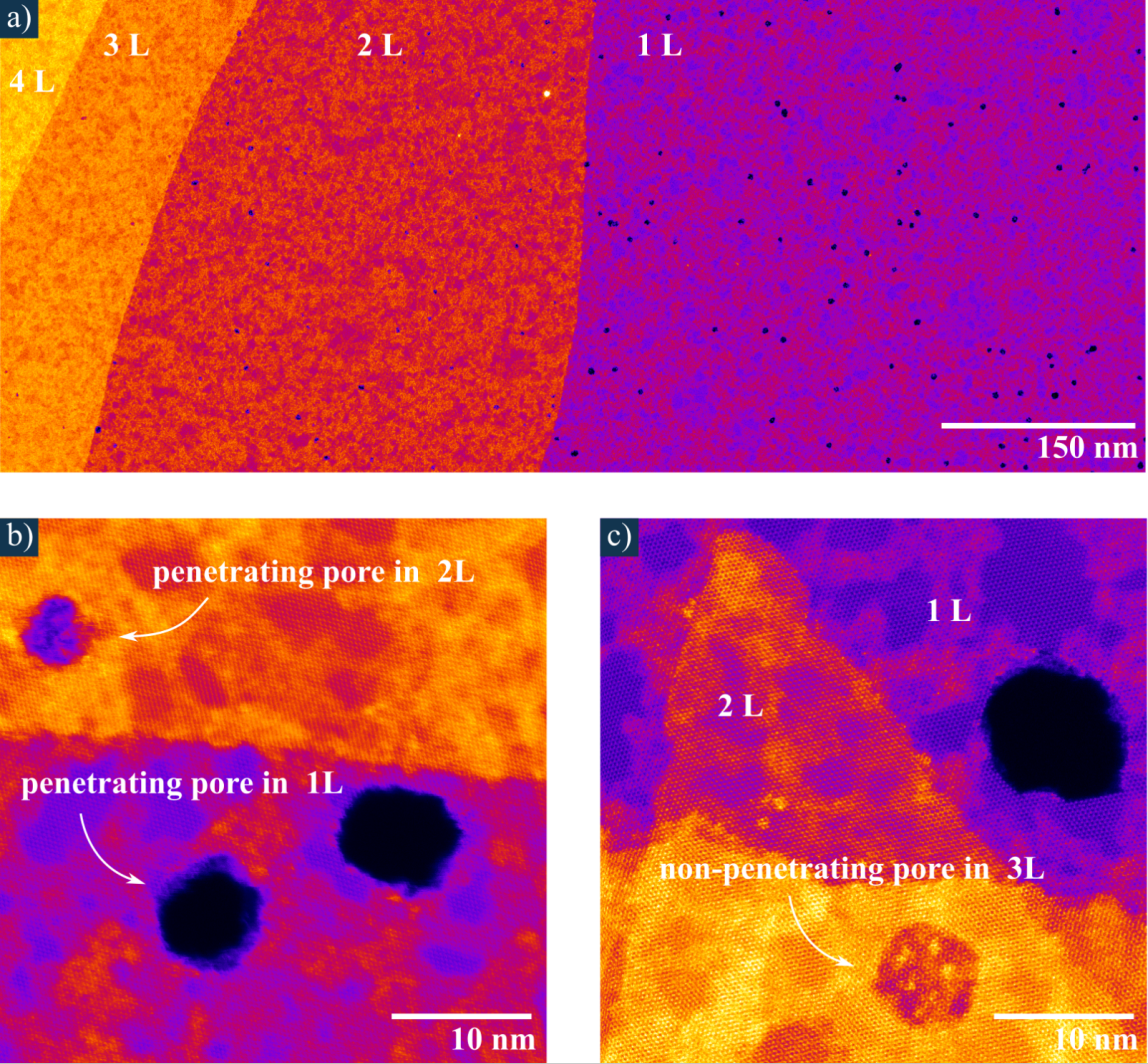
(a) STEM ADF images of MoS_2_ on SiO_2_ with different layer numbers, irradiated with 180 keV Xe^37+^ ions. (b) Fewer and smaller pores are observed in bilayer MoS_2_ (2L) compared to monolayer on SiO_2_ (1L). (c) In trilayer MoS_2_, pores are predominantly non-perforating.

To test the role of out-of-plane electronic energy dissipation, we exploit regions of different thickness in MoS_2_ grown via chemical vapor deposition (CVD) on SiO_2_ (Figure 2a). Compared to monolayer regions (1L), bilayer MoS_2_ (2L) shows both reduced pore radii and reduced pore formation efficiency (Figure 2b). In trilayer regions (3L), fully perforating pores are strongly suppressed and partially perforating (non-through) pores occur (Figure 2c). These observations indicate that out-of-plane electronic energy dissipation is present but limited in magnitude: Coupling to a second layer is sufficient to modify pore formation, yet the available excitation appears insufficient to efficiently drive a fully perforating defect through three layers.

The observed anisotropy of the defects (radius ≈2.2 nm; depth ≈1.5 nm) likely reflects the strongly different in-plane and out-of-plane dissipation pathways in layered MoS_2_ [50–51], which disfavor spherical damage volumes.

### Swift heavy ions

**Figure 3:**
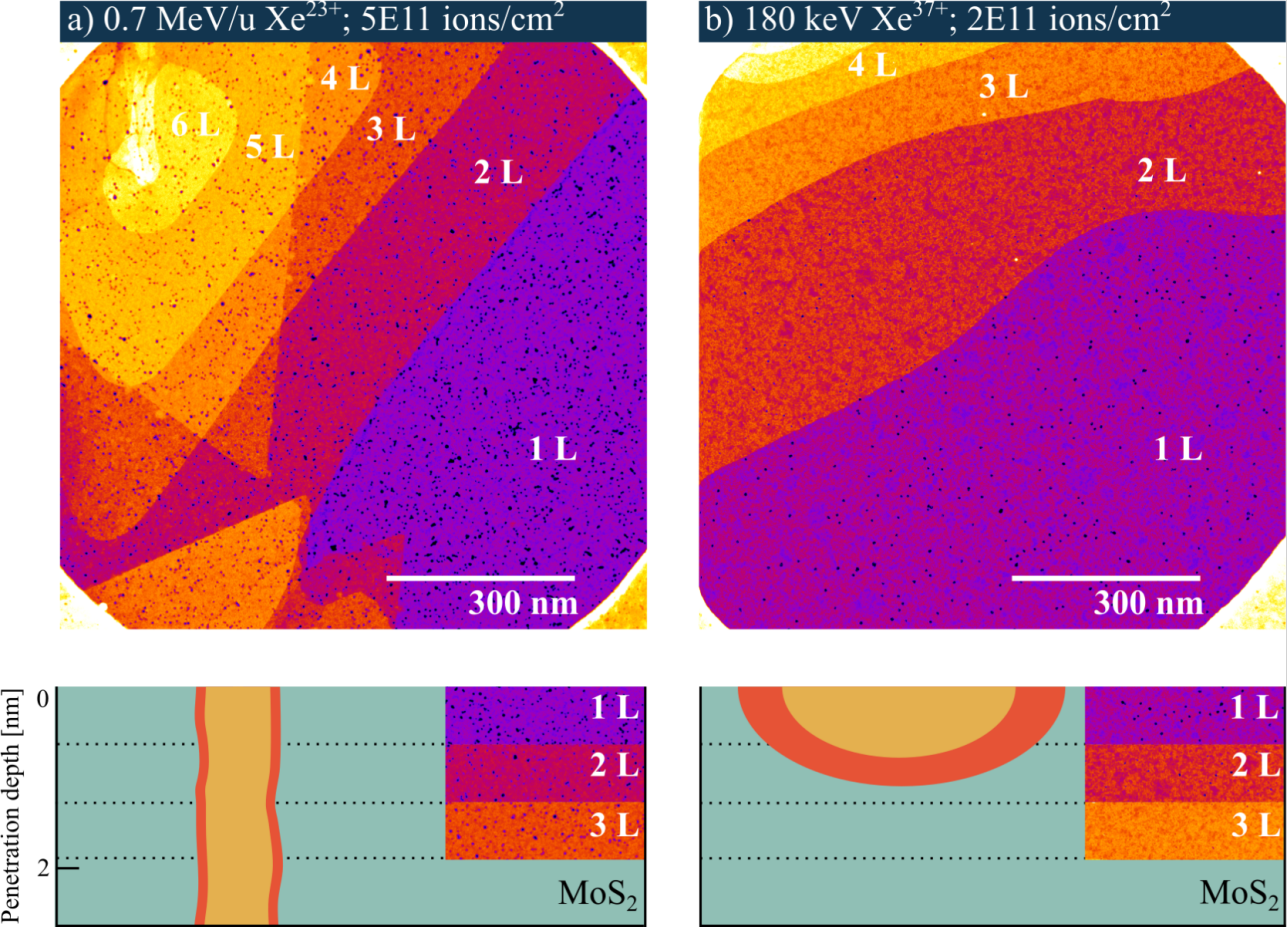
STEM ADF images of SHI-irradiated (a) and HCI-irradiated (b) MoS_2_ reveal similar pore dimensions in 1L, but, as expected, vastly different perforation behavior. Schematics below each image illustrate electronic energy deposition profiles of the different irradiation types. For HCIs, the schematic does not represent the full ion trajectory. These illustrations are visual representations and not to scale.

In addition to HCI irradiation, we investigate samples irradiated with SHIs. SHIs deposit energy continuously along their trajectory via electronic stopping *S*_e_, producing excitation and ionization of the target’s electronic system [52–53] (Figure 3). This extended deposition profile provides a complementary probe of substrate-controlled electronic energy dissipation. Based on previous results on suspended MoS_2_, the SHIs used in this study are expected to deposit in the order of ≈10 keV (0.7 MeV/u Xe^23+^) and ≈20 keV (4.8 MeV/u Au^25+^) in a monolayer [13].

**Figure 4:**
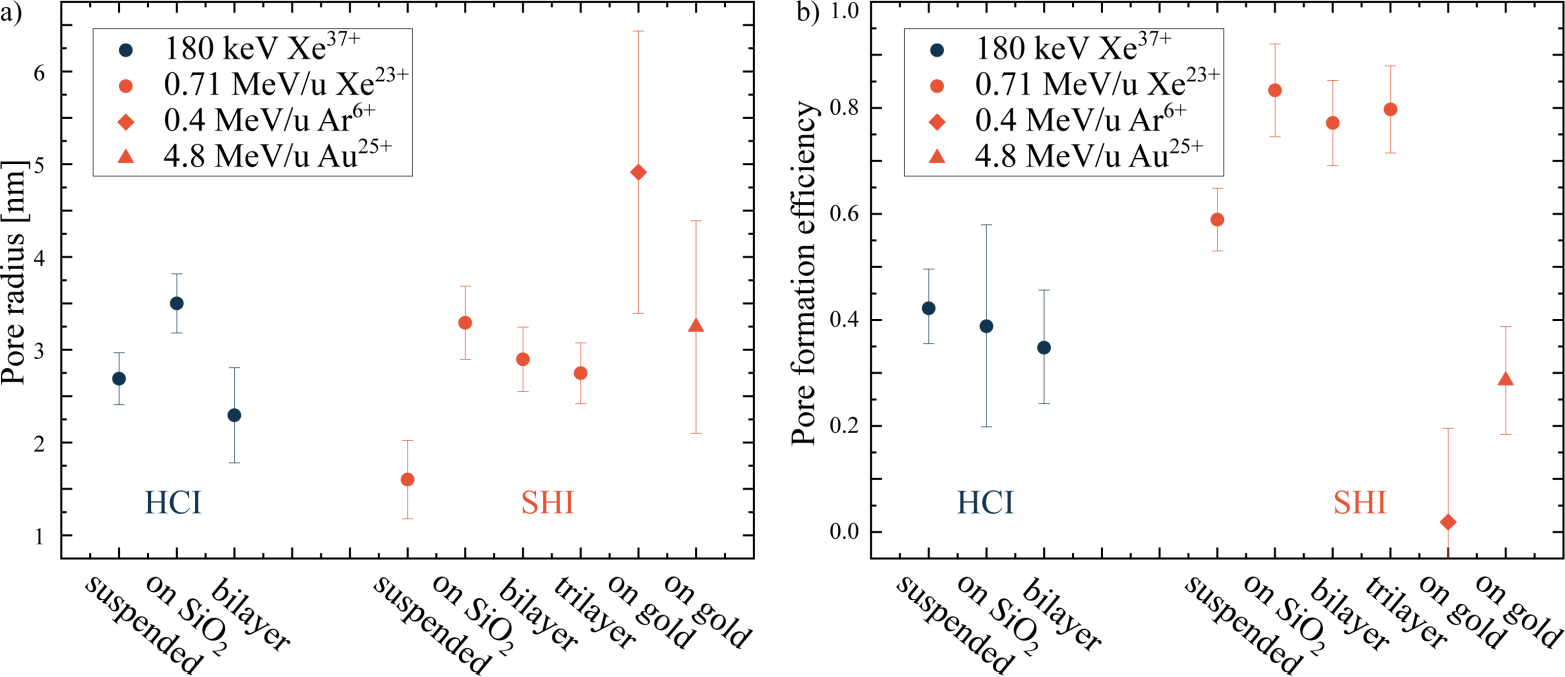
Mean pore radii (a) and pore formation efficiencies (b) of HCI- (blue) and SHI-irradiated MoS_2_ (red) in different configurations. Pore formation efficiency is measured using overview STEM images containing ≈100 pores (see Supporting Information File 1). Error bars of mean pore radii are the standard deviation, while uncertainty in efficiency arises from pore counting and fluence determination in all cases. For HCI-irradiated supported samples, additional contributions arise from partial-area irradiation due to transfer-related misalignment (see Supporting Information File 1, Figure S3) and from the contamination-related effective-area estimate discussed in Supporting Information File 1.

In Figure 3, representative multilayer regions after HCI and SHI irradiation are shown. Pores in monolayers are of comparable size, while the perforation behavior differs strongly. Quantitative pore radii and pore formation efficiencies, defined here as the number of pores formed per incident ion, are summarized for all configurations in Figure 4. For both ion types, monolayer MoS_2_ on SiO_2_ exhibits the largest pores. However, thickness affects the two regimes differently: Under SHI irradiation, pore size and efficiency depend only weakly on the layer number, whereas HCI-induced pore formation attenuates rapidly with thickness. This contrast is consistent with SHIs transferring electronic energy along an extended track deep into the target, while HCIs release their potential energy predominantly near the surface during a single neutralization event.

The pronounced difference in size and efficiency between suspended and supported MoS_2_ under SHI irradiation is consistent with the strong impact-parameter dependence of electronic energy deposition in an atomically thin target [13]. In SHI-irradiated suspended monolayers, a fraction of ion impacts generate local energy densities below the threshold required for pore formation, depending on the trajectory relative to atomic sites. Adding a substrate and/or additional layers reduces the likelihood of such “sub-threshold” events because energy deposition and subsequent dissipation are distributed across coupled layers. Moreover, we previously showed [13] that pore formation under SHI irradiation is strongly reduced in suspended membranes because part of the deposited energy is carried away by atoms and ions emitted from both sides of the membrane before it can be converted into local lattice heating. This lowers the energy retained near the ion impact and therefore reduces the probability of pore formation. For HCIs, the initial interaction is governed by near-surface charge exchange within a nanometer-scale zone spanning multiple atoms [54–55]. This may reduce sensitivity to atomic-scale impact parameter compared to the SHI impact.

For HCI-irradiated supported samples, additional contributions arise from partial-area irradiation due to transfer-related misalignment (see Supporting Information File 1, Figure S3) and from the contamination-related effective-area estimate discussed in Supporting Information File 1. We observe that the pore formation efficiencies differ for the two ion types. HCIs yield generally lower efficiencies (η ≈ 0.4) for the same target configuration than SHIs (η ≈ 0.6–0.9). However, the HCI efficiencies carry larger systematic uncertainty and should therefore be interpreted with caution. These uncertainties arise from the fluence gradient across the beam spot, transfer-related misalignment (Supporting Information File 1, Figure S3) and fluence determination. An additional factor affecting the local efficiency may be hydrocarbon contamination. Ambient exposure produces hydrocarbon coverage on 2D materials [56], and we observe indications of locally reduced pore formation in contaminated regions (Supporting Information File 1, Figure S5), which likely affects the apparent efficiency in the case of HCI. A more precise determination of the HCI efficiency likely requires dedicated in situ experiments. Within the uncertainty of the present dataset, no clear substrate dependence of the HCI pore-formation efficiency can be resolved.

For SHI irradiation, the efficiency data are less affected by these specific uncertainties. Efficiency in supported MoS_2_ approaches saturation (η ≈ 0.8), whereas efficiency in the suspended monolayer remains well below saturation η ≈ 0.6. This behavior can, again, be traced back to sub-threshold energy transfer impacts that can occur in monolayers [13]. The notably low efficiency observed for MoS_2_ on Au (η ≈ 0.28) stands apart from this trend and will be discussed further below.

A remaining question is whether pore formation can be mitigated by the right choice of substrate. Schwestka et al. [28] demonstrated that graphene/MoS_2_ heterostructures can be resistant to HCI-induced pore formation when MoS_2_ is covered by graphene as charge exchange and potential energy deposition primarily occur within graphene. Importantly, when the heterostructure is inverted such that the HCI reaches MoS_2_ first, pores are still created in MoS_2_. This behavior is consistent with the weak coupling of different layers in van der Waals heterostructures. Although graphene is an excellent in-plane conductor, the out-of-plane electrical (and thermal) coupling across the graphene/MoS_2_ interface is comparatively weak [57–58]. Indeed, simulations by Schwestka et al. [28] suggest that the out-of-plane conductance across the graphene/MoS_2_ interface is two orders of magnitude smaller than the in-plane conductance of the individual layers. Consistent with limited charge transfer between MoS_2_ and graphene, no strong PL quenching through loss of photoexcited charge carriers from the MoS_2_ into the graphene is observed in graphene/MoS_2_ heterostructures [59].

In contrast, coupling between 2D semiconductors and Au can be substantially stronger, which underlies the success of Au-assisted exfoliation and the formation of large-area, clean interfaces [60–61]. Motivated by this, we irradiated MoS_2_ exfoliated on Au to test whether a metallic substrate with strong interfacial coupling can act as an efficient sink for transient electronic excitation and charge, thereby suppressing pore formation under electronically dominated ion impact. In contrast to the samples discussed above, for which MoS_2_ was transferred after irradiation using only water, transfer from Au substrates to TEM grids requires chemical removal of the gold support using an iodine/iodide etchant (KI/I_2_). Consequently, unlike in the previous case, the ion-induced pores cannot be assumed to remain unaffected during transfer [62], as they were in the previous case. For this reason, the pore formation efficiency is expected to be the more reliable observable for a quantitative discussion of this substrate, whereas pore dimensions can only be discussed qualitatively.

**Figure 5:**
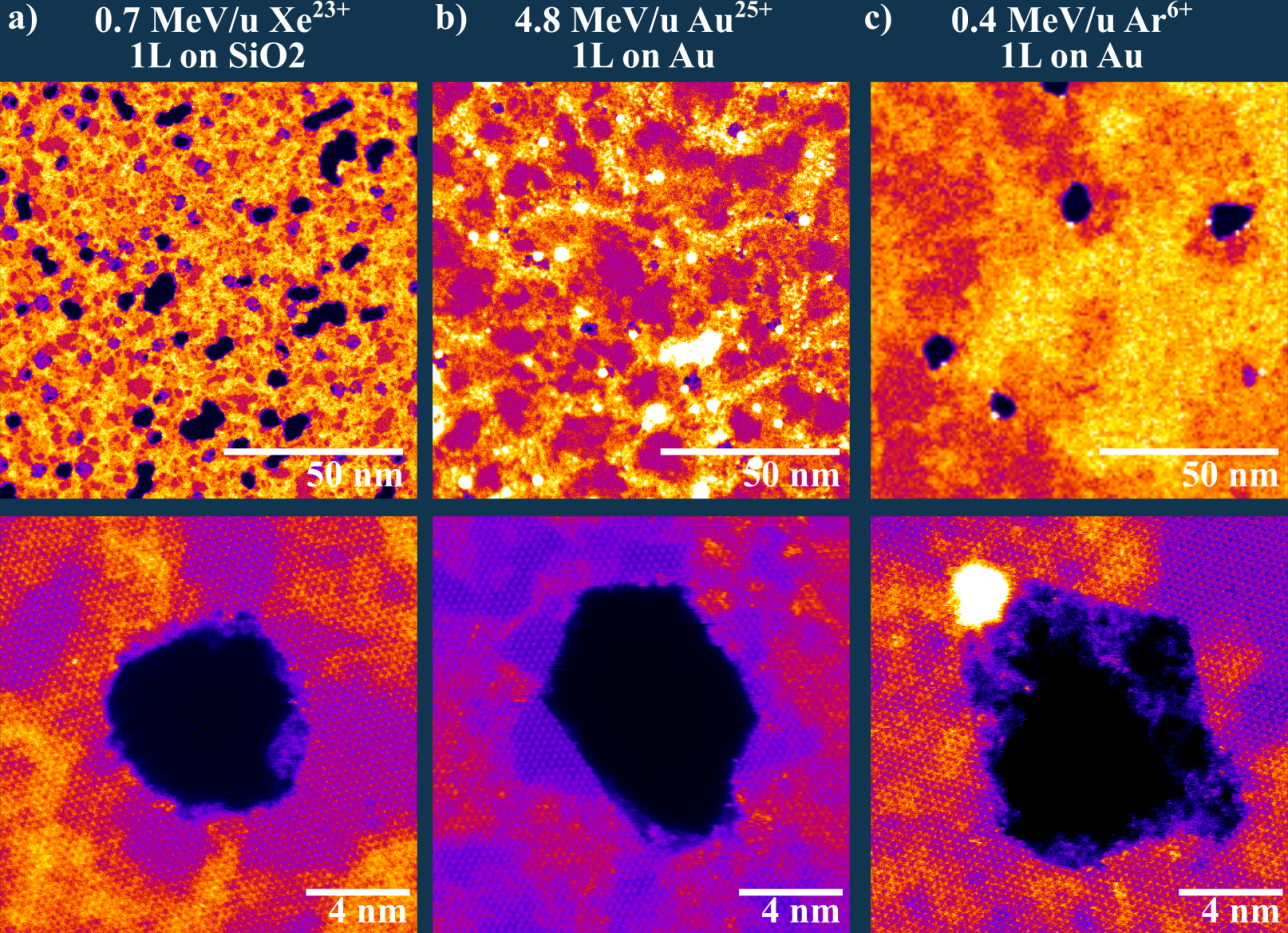
STEM ADF images of monolayer MoS_2_ irradiated on SiO_2_ (a) and on Au substrates (b, c). Based on estimates for suspended MoS_2_ and neglecting substrate effects, the three irradiations are expected to deposit approximately 10 keV in (a), 20 keV in (b), and 5 keV in (c) per monolayer [13]. The observed pore sizes do not follow this expected trend with deposited energy. In addition, pores formed on Au show unusual morphologies and a broad size distribution. Despite the presumably larger deposited energy in (b) than in (a), the pore formation efficiency on Au is strongly reduced. Note that all displayed samples received the same fluence.

Figure 5 compares SHI-irradiated MoS_2_ on SiO_2_ (Figure 5a) with MoS_2_ irradiated on Au (Figure 5b,c). The three irradiations use different ions, whose mean energy transfer 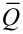 to the monolayer can be estimated from results for suspended MoS_2_ [13]: 4.8 MeV/u Au^25+^ (Figure 5b) is expected to transfer about 20 keV per sheet, 0.71 MeV/u Xe^23+^ (Figure 5a) about 10 keV per sheet, and 0.4 MeV/u Ar^6+^ (Figure 5c) about 5 keV per sheet. Neglecting substrate and transfer-related effects, one would therefore expect the average pore size to decrease from Figure 5b to Figure 5a to Figure 5c. Instead, the mean pore radii extracted in Figure 4a show that pores formed on Au under 4.8 MeV/u Au^25+^ irradiation are comparable in size to those on SiO_2_, while the 0.4 MeV/u Ar^6+^ irradiation of MoS_2_ on Au yields on average the largest pores. The pores on Au additionally exhibit a broad size distribution and an unusual morphology (see Supporting Information File 1, Figure S6 and Figure S7), with many showing straight, lattice-aligned edges consistent with faceted etch pits [63]. We interpret this as evidence of the aforementioned influence of the etchant during transfer, which may bias the measured pore sizes toward larger values [64].

All irradiations shown in Figure 5 were performed at the same fluence (5 × 10^11^ cm^−2^). The pore formation efficiency of MoS_2_ on Au in Figure 5b is strongly reduced (η ≈ 0.28) compared with MoS_2_ on SiO_2_ in Figure 5a (η ≈ 0.83), despite the higher expected energy transfer for the Au irradiation. For the low-energy SHI irradiation (Figure 5c), efficiency is further reduced to η ≈ 0.02. This behavior is consistent with the hypothesis that out-of-plane dissipation of electronic excitation into the Au substrate reduces the probability of pore formation. One possible mechanistic interpretation is that coupling to Au effectively raises the pore-formation threshold in MoS_2_ relative to MoS_2_ on SiO_2_. As shown previously [13], even modest threshold shifts can strongly affect the pore formation efficiency.

The inferred efficient out-of-plane dissipation from MoS_2_ into Au is consistent with the well-known strong MoS_2_–Au interfacial coupling. Our own Raman and PL characterization of the MoS_2_/Au samples (see Supporting Information File 1, Figure S2) shows splitting of the out-of-plane *A*_1_*_g_* mode and strong PL quenching relative to MoS_2_ on SiO_2_, indicating that the interface modifies both lattice dynamics and carrier recombination in the as-prepared samples. The microscopic origin of this coupling has been attributed to Fermi-level pinning arising from interface–dipole formation and the appearance of Mo d-orbital gap states due to metal–S interactions at the interface [61,65]. Whether these equilibrium coupling mechanisms directly govern the efficiency of transient charge transfer under ion impact remains an open question, but the consistency between the strong equilibrium coupling and the observed suppression of pore formation supports the interpretation that Au provides an efficient dissipation channel for electronically driven damage.

## Conclusion

We have shown that substrate coupling plays a decisive role in electronically driven pore formation in MoS_2_ under both HCI and SHI irradiation. Compared with suspended membranes, an insulating substrate increases pore size, additional out-of-plane dissipation in MoS_2_ multilayers suppresses HCI-induced pore formation, and a strongly coupled metallic substrate (Au) markedly reduces the pore formation efficiency for SHI irradiation. Together, these trends indicate that, for ions that primarily interact with the electronic system of the target, pore evolution is governed less by the energy deposition mechanism than by how efficiently that electronic excitation is redistributed and converted into lattice motion under the constraints imposed by the interface. The trends in pore size and pore formation efficiency observed here are robust, but the underlying dissipation pathways are inferred indirectly from defect statistics rather than measured directly. This limitation does not alter the main comparative conclusions, but it does constrain the level of mechanistic interpretation that can be drawn from the present dataset. Nevertheless, the substrate- and thickness-resolved pore statistics presented here provide quantitative benchmarks for future modeling. A particularly promising next step would be a targeted description of electronic energy dissipation across the interface between the 2D material and the substrate.

## Experimental

### Sample preparation

**Figure 6:**
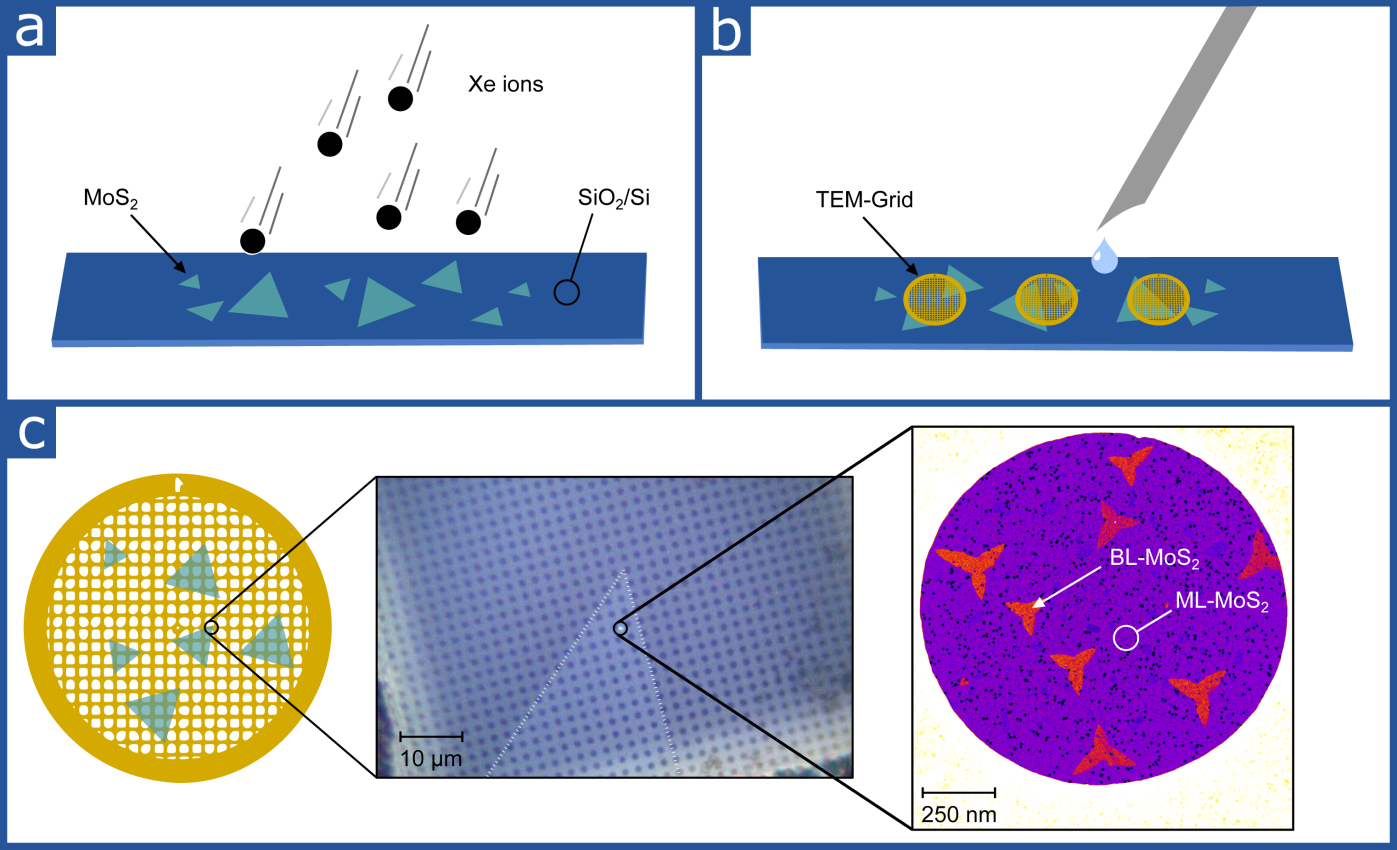
Schematic of the irradiation and subsequent STEM analysis of MoS_2_ on SiO_2_. First, CVD-grown MoS_2_ on SiO_2_/Si is irradiated with highly charged ions (a). After that, irradiated MoS_2_ is transferred polymer-free to a TEM grid (b). (c) Transferred flakes on the TEM grid (left) are visible in a light microscope (center). On the right, a STEM ADF image of a single TEM grid pore covered by monolayer, and occasionally bilayer, MoS_2_ is shown.

A custom chemical vapor deposition (CVD) process is used to grow monolayer MoS_2_. Substrates are prepared by placing a micro-droplet of saturated ammonium heptamolybdate onto a 300 nm SiO_2_/Si substrate. The substrate is then spin-coated with a 1% sodium cholate solution that acts as a growth promoter. Such prepared substrates are then placed into the second zone of a three-zone furnace. In the first zone ≈60 mg of sulfur is placed. An argon flow of 500 sccm is used to purge the quartz tube containing the reagents and as a means to transport the precursors. During the 30 min process, the first zone reaches 150 °C, while the second zone reaches 750 °C. After the process has finished, the furnace is opened to allow for rapid cooling. The freshly grown MoS_2_ flakes are irradiated on the SiO_2_/Si substrate and subsequently transferred to a QUANTIFOIL^®^ holey carbon grid with 1.2 µm holes. The transfer is performed by placing the TEM grid onto the sample with the mesh facing the MoS_2_. By placing a droplet of water near the grid, the water can intercalate between the flakes and the substrate, lifting off the MoS_2_ in the process and pressing it against the QUANTIFOIL^®^ holey carbon mesh. Sample preparation and characterization are schematically shown in Figure 6.

To investigate the irradiation of MoS_2_ on Au, a substrate suitable for mechanical exfoliation is first prepared. A 5 nm Ti adhesion layer is deposited onto SiO_2_, followed by the deposition of a 25 nm Au film. Immediately after deposition, MoS_2_ is mechanically exfoliated onto the freshly prepared Au surface.

After irradiation on Au, the monolayers have to be transferred onto TEM grids for STEM characterization. For this purpose, a thin polystyrene (PS) layer is spin-coated onto the sample. The sample is then immersed in a preheated KI/I_2_ etching solution (4 g KI, 1 g I_2_, 150 mL H_2_O) at 40 °C. After approximately 1 h, the Au layer has dissolved, allowing the PS film with the attached monolayers to be retrieved and cleaned in ultrapure water.

The film is then transferred onto a TEM grid and heated for 30 min at 80 °C, followed by 1 h at 130 °C. During this step, slow heating and cooling are essential to avoid damaging the TEM grid. Finally, the PS film is dissolved in toluene for 2 h, with the solvent renewed once during the process. After retrieval, the grid is immersed in analytical-grade isopropanol as a final cleaning step and then left to dry.

### Ion irradiation

Irradiation with highly charged xenon ions was done at the HICS beamline at the University of Duisburg-Essen [42,66–67]. The ions with charge states *q* = 28+ up to *q* = 44+ are provided by an electron beam ion source (EBIS). Samples were irradiated under perpendicular incidence with a fluence of Φ = 5 × 10^11^ cm^−2^. At this fluence, the density of pores is high enough to image them efficiently in the STEM, while it is low enough to avoid frequent occurrence of overlapping pores (see Supporting Information File 1). Irradiation was performed at three different kinetic energies (20, 180, and 260 keV) in order to evaluate the influence of the kinetic energy on the pore creation. As there was no evidence for differences between the two high energies, no additional irradiations at 260 keV after the initial one were done. Irradiation with SHIs was performed at IRRSUD at GANIL, Caen (0.4 MeV/u ^36^Ar^6+^; 0.7 MeV/u ^129^Xe^23+^) and at M-Branch at GSI, Darmstadt (4.8 MeV/u ^197^Au^25+^). For both irradiations, the flux was kept below 1 × 10^9^ s^−1^·cm^−2^ to avoid thermal damage. Total fluence was again set to Φ = 5 × 10^11^ cm^−2^. All irradiations were under perpendicular incidence.

### Imaging

STEM measurements were carried out in Vienna with an aberration-corrected Nion UltraSTEM 100. Prior to imaging, the samples were heated at 170 °C for around 10 h in vacuum to remove water and minimize the amount of surface contamination, and inserted into the microscope without air exposure [68]. The images were recorded with a medium-angle annular dark-field (MAADF) detector with a collection semiangle of 60–200 mrad. The acceleration voltage of electrons was 60 kV, and a dwell time of 8 µs/px and a flyback time of 120 µs were used to record images with 1024 × 1024 px. Images were recorded at fields of view of 32 nm for the pore size analysis and 512 nm for obtaining an overview. At least 150 pores were analyzed for each set of ion irradiation parameters. Pore sizes were determined from the STEM images by manually evaluating the projected pore area. The area was then converted into an equivalent circular pore radius *r* according to *A* = π*r*^2^. This area-based definition was used because it does not require the pores to be perfectly circular, which is important since deviations from an ideal round shape are observed for some pores. Manual area evaluation was chosen to remain consistent with our previous study on suspended layers and because contamination-induced contrast variations can make purely threshold-based edge detection unreliable. To ensure comparability with the suspended-layer reference data, which originate from our previous work, the same general procedure regarding sample preparation, STEM imaging, and image analysis was applied.

### Raman and PL spectroscopy

Raman and PL spectroscopy were performed using a WITec alpha300 RA confocal Raman spectrometer. In all instances, a green laser (λ = 532 nm) with a maximum power of 1 mW was used. For Raman spectra, a 1800 L·mm^−1^ grating was used, while a 300 L·mm^−1^ grating was used for PL recording.

## Supporting Information

File 1 Additional experimental results.

## Data Availability

Data generated and analyzed during this study is openly available in Zenodo at https://doi.org/10.5281/zenodo.19482837.
