# Supplementary material for: Substrate-dependent pore formation in molybdenum disulfide monolayers under ion irradiation
Source: Beilstein J Nanotechnol. 2026 Jun 12;17:769–80. doi: 10.3762/bjnano.17.54 (PMC13267488; doi:10.3762/bjnano.17.54)
Supplement: File 1 — Additional experimental results. [file Beilstein_J_Nanotechnol-17-769-s001.pdf]

BEILSTEIN JOURNAL OF NANOTECHNOLOGY

Supporting Information

for

Substrate-dependent pore formation in molybdenum disulfide monolayers under ion irradiation

Yossarian Liebsch, Umair Javed, Lucia Skopinski, Leon Daniel, Franziska Appel, Radia Rahali, Clara Grygiel, Henning Lebius, Carolin Frank, Lars Breuer, Leon Kirsch, Frieder Koch, Jani Kotakoski and Marika Schleberger

Beilstein J. Nanotechnol. **2026,** *17,* 769–780. doi:10.3762/bjnano.17.54

Additional experimental results

License and Terms: This is a supporting information file under the terms of the Creative Commons Attribution License (https://creativecommons.org/licenses/by/4.0). Please note that the reuse, redistribution and reproduction in particular requires that the author(s) and source are credited and that individual graphics may be subject to special legal provisions.
The license is subject to the *Beilstein Journal of Nanotechnology* terms and conditions: (https://www.beilstein-journals.org/bjnano/terms)

Strain around pores

The strain around selected pores was analyzed by geometrical phase analysis (GPA) using the open-source software Strain++. Fig. S1 shows a representative STEM ADF image and the corresponding in-plane strain-tensor components. Within the resolution of this analysis, no obvious long-range strain field is resolved around the pore shown.

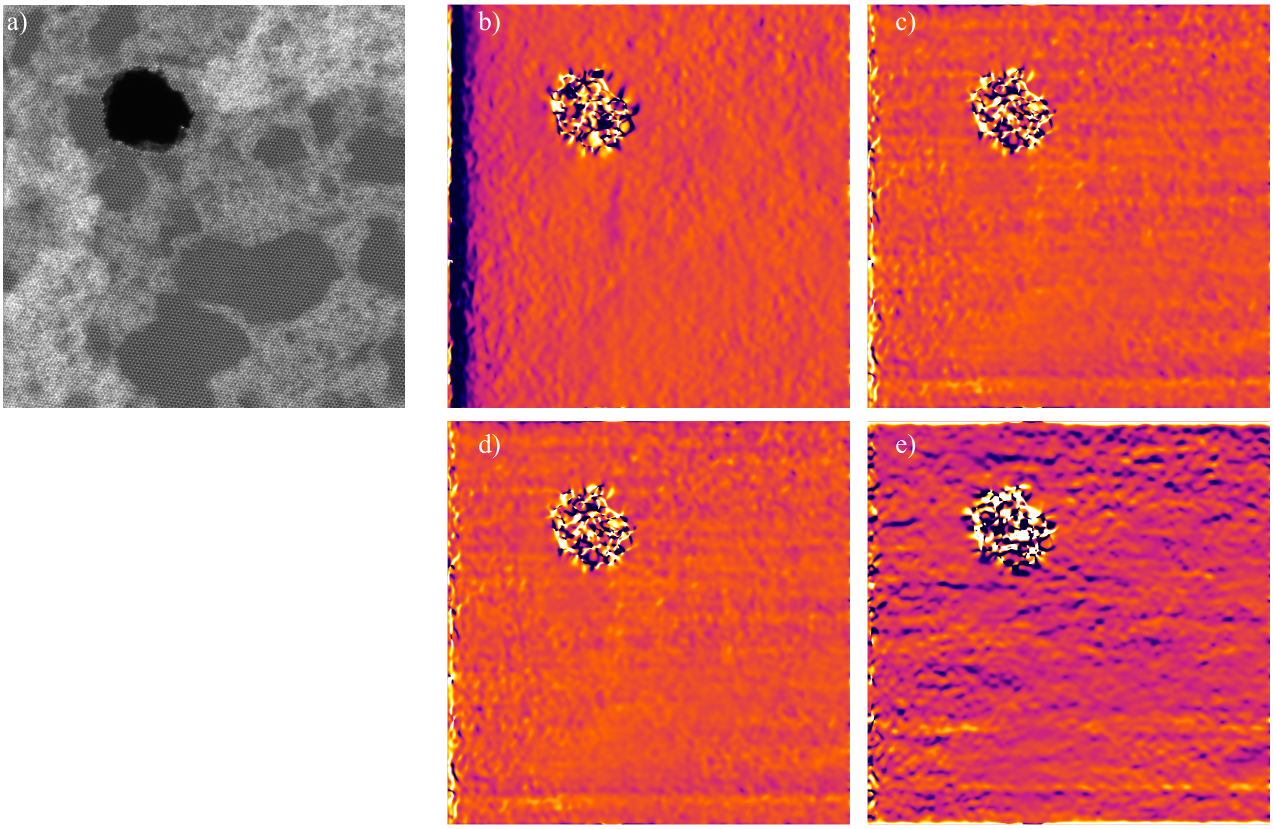

Figure S1: Representative STEM ADF image of a pore (a) and the corresponding GPA strain components: ϵ_{xx}(b), ϵ_{xy}(c), ϵ_{yx}(d) and ϵ_{yy}(e). No obvious long-range strain field is resolved around this pore within the sensitivity of the analysis.

Raman and PL measurements

Results of Raman and PL measurements of suspended MoS_2, MoS_2 on SiO_2 and MoS_2 on Au are given in Fig. S2. The A_{1g} Raman mode of MoS_2 on Au (a) exhibits the characteristic splitting associated with strong MoS_2–Au interaction. In addition, PL of MoS_2 on Au is strongly quenched

relative to suspended MoS_2 and MoS_2 on SiO_2, consistent with efficient nonradiative relaxation and/or charge transfer at the MoS_2–Au interface.

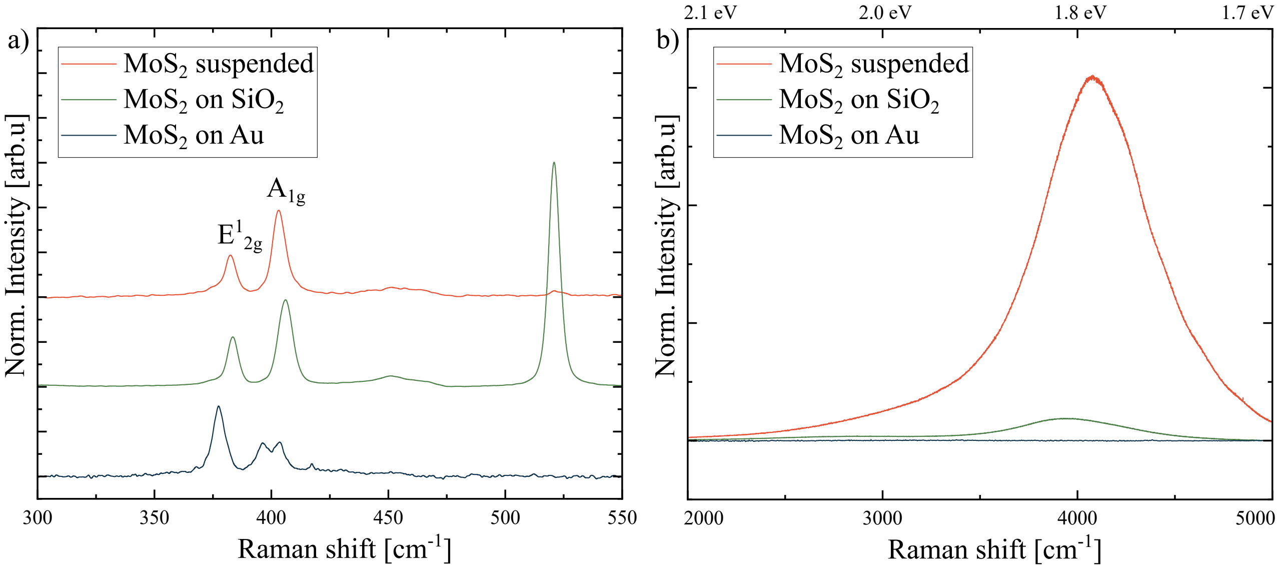

Figure S2: Raman (a) and PL (b) spectra of MoS_2 on SiO_2, on gold and suspended. Due to the strong coupling with the gold substrate, the out-of-plane A_{1g} mode exhibits a characteristic splitting. In addition, charge carrier dissipation into the substrate quenches the PL completely.

General information on efficiency

Pore formation efficiency η is defined as the number of pores per incident ion,

$$\eta = \frac{N_{pore}}{N_{ion}} = \frac{N_{pore}}{\Phi A}, \tag{S1}$$

where N_{pore} is the number of pores identified in the analyzed STEM image area A and Φ is the fluence. The incident fluence was determined from beamline diagnostics, including Faraday cup measurements. Depending on the irradiation facility, the absolute fluence may deviate from the nominal value by approximately 5–10%. In addition, the finite number of counted pores contributes a statistical uncertainty. Assuming Poisson counting statistics, $\Delta N_{pore} \approx \sqrt{N_{pore}}$, giving a relative counting uncertainty of approximately $1/\sqrt{N_{pore}}$. Accordingly, data points with lower efficiency, and therefore smaller pore counts within a fixed image area, generally exhibit larger relative uncertainty.

This expression accounts for pore-counting statistics and the uncertainty of the beamline fluence measurement, but does not include the additional systematic uncertainty discussed below for partially irradiated HCI-supported samples.

The probability of two or more ion impacts occurring within the area associated with a single pore (here referred to as “missing impacts”) was estimated as

$$P = 1 - e^{-\Phi A_{\text{eff}}} \left(1 + \Phi A_{\text{eff}}\right), \tag{S2}$$

where Φ is the ion fluence and A_{eff} is the effective pore area. This expression gives the probability that two or more impacts occur within the same area A_{eff}, assuming a spatially random Poisson distribution of ion impacts. Here, A_{eff} denotes an approximate indistinguishability area, i.e., the area within which two nearby impacts can no longer be reliably separated in the image analysis. Because this threshold is not sharply defined, the estimate should be understood as order-of-magnitude only. Using the experimentally observed pore sizes and a conservative minimum separable spacing on the order of the pore diameter, we obtain $P < 5\%$ for all investigated configurations at $\Phi = 5 \times 10^{11}$ cm^{-2}. Pore size analysis was restricted to isolated defects that could be assigned to single impacts.

Spatial variations of efficiency

For substrate-supported samples irradiated with HCIs, only a limited region of the macroscopic substrate could be exposed because the available ion flux decreases strongly at high charge state. In contrast, SHI irradiations covered the full substrate area. After irradiation, the supported MoS_2 had to be transferred to a TEM grid for STEM characterization. This transfer is prone to lateral misalignment and local membrane displacement, such that the analyzed region on the TEM grid does not necessarily coincide with the center of the irradiated area. As a result, the apparent pore-formation efficiency can vary spatially across a transferred grid, whereas the pore radius is expected to be much less sensitive to this effect. Fig. S3 illustrates the geometric origin of this systematic uncertainty, and Fig. S4 shows a representative position-resolved efficiency map for one sample. Each local value was obtained by

averaging five STEM images with fields of view of approximately 500×500 nm^2, each containing on the order of 100˘200 pores. For these partially irradiated HCI-supported samples, the maximum local average efficiency measured on a grid is taken as the best estimate of the peak efficiency, and the uncertainty shown in the main manuscript includes the corresponding local standard deviation together with the fluence and counting uncertainties discussed above.

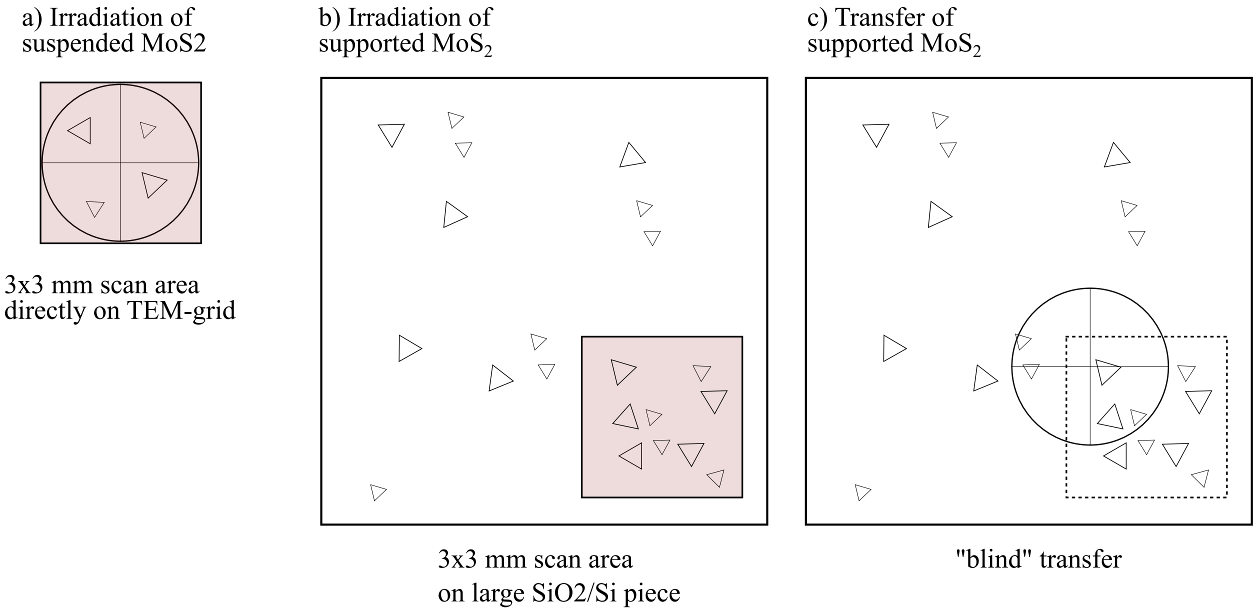

Figure S3: Schematic of the systematic error arising from irradiating a small area on a sample and subsequently transferring it to a TEM grid. Misalignment and movement of the single-layers can offset the center beam position on the TEMgrid.

Influence of contamination

Surface contamination is an important experimental factor in this work. Samples were not annealed prior to irradiation; instead, annealing was performed only before STEM imaging. This protocol was chosen for consistency across the dataset, but it implies that irradiation occurred in the presence of varying amounts of adsorbates and hydrocarbon contamination. Especially for HCI irradiation, such surface layers may influence charge exchange between the projectile and the two-dimensional target and may therefore contribute to scatter in the measured local pore-formation efficiency. The results presented here should therefore be interpreted as ensemble trends obtained under identical

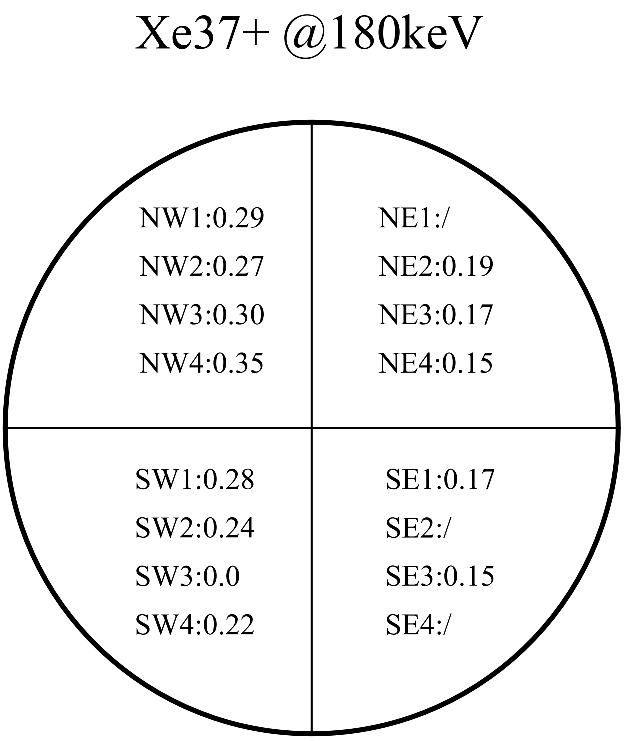

Figure S4: Representative position-resolved pore-formation efficiency measured across a transferred TEM grid for the 180 keV $Xe^{37+\cdot}$ irradiation. The highest local average efficiency is observed in the north-west region, consistent with imperfect overlap between the irradiated area and the transferred membrane.

sample-handling conditions, while contamination remains a possible secondary contributor to the absolute efficiencies.

In the post-annealing STEM images, pores are rarely observed in atomically clean regions. At least two non-exclusive explanations are plausible. First, contamination present during irradiation may partially shield the MoS_2 and later be removed during annealing, leaving apparently clean regions that experienced a reduced effective exposure. Second, contamination may preferentially accumulate at pore edges after irradiation because undercoordinated atoms at the rim act as adsorption sites. Irradiation-induced modification or removal of surface contamination may further complicate this picture. Accordingly, the present dataset does not allow the direction of causality to be established. Dedicated experiments with controlled in situ cleaning before irradiation would be required to separate these effects.

To obtain a first quantitative estimate of the spatial correlation between pores and contamination,

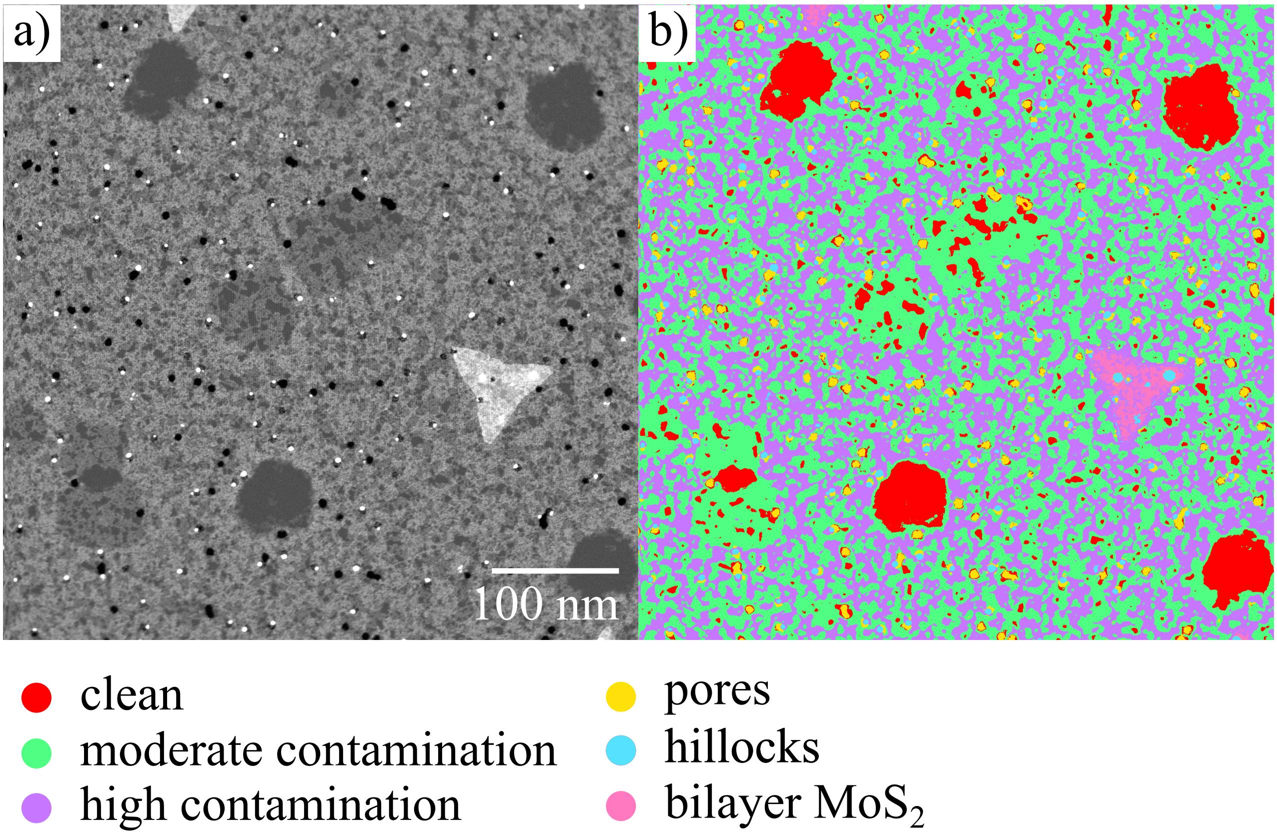

Figure S5: Large-field-of-view STEM ADF image of an HCI-irradiated MoS_2 monolayer (a) and the corresponding segmented image (b). Classification is based primarily on image contrast and feature size and is shown here to illustrate the spatial correlation between pores and contamination.

representative STEM images were segmented in ImageJ using the Trainable Weka Segmentation plugin into the classes clean, moderate contamination, high contamination, pores, dots, and bilayer MoS_2. Within this classification, pores were found predominantly in regions labeled as moderate contamination. Because the result depends on image contrast and classifier settings, we use it here only as a qualitative indication rather than a precise quantitative measure.

To estimate the possible magnitude of this effect on the pore-formation efficiency, we performed a conservative sensitivity analysis. In the nominal analysis, the full evaluated MoS_2 image area was used to calculate the efficiency according to $\eta = N_{\mathrm{pore}}/(\Phi A)$. As an upper-bound estimate, we recalculated the efficiency after excluding apparently clean, pore-free regions from the evaluated image area. This limiting assumption corresponds to the case in which these regions did not effectively contribute to pore formation, for example because they were shielded during irradiation.

For the analyzed HCI-supported dataset, the apparently clean, pore-free regions account for approximately 7% of the evaluated image area. Excluding this area reduces the effective area to $A_{\mathrm{eff}} = 0.93A$ and therefore increases the efficiency by a factor of $1/0.93$, leading to a systematic upward shift of the efficiency. The contamination-related effective-area contribution changes the uncertainty only slightly for this dataset. Nevertheless, it is included as an asymmetric upper-bound contribution to the HCI efficiency uncertainty in Fig. 4b of the main manuscript.

Pore shape on different substrates

As noted in the main manuscript, pores formed in suspended monolayer MoS_2 and in MoS_2 on SiO_2 are generally close to circular. In contrast, for MoS_2 irradiated on Au, a considerable fraction of pores—especially the larger ones—deviates from circularity. This behavior was observed in two independent irradiations: the (4.8 MeV/u Au^{25+}) dataset discussed in the main manuscript and an additional lower-energy irradiation (0.4 MeV/u Ar^{6+}) shown here. In both cases, many pores exhibit straight, lattice-aligned edges. The lower-energy Au-supported dataset shows the largest apparent pore sizes despite the lowest pore-formation efficiency (η ≈2%). This supports the view that the pore morphology on Au is modified during transfer and Au removal, most likely by the KI/I_2 etching step, whereas the pore-formation efficiency is the more robust observable for comparing irradiation-induced damage across substrates. Fig. S6 shows representative close-up STEM images together with larger-field-of-view images illustrating the corresponding defect densities.

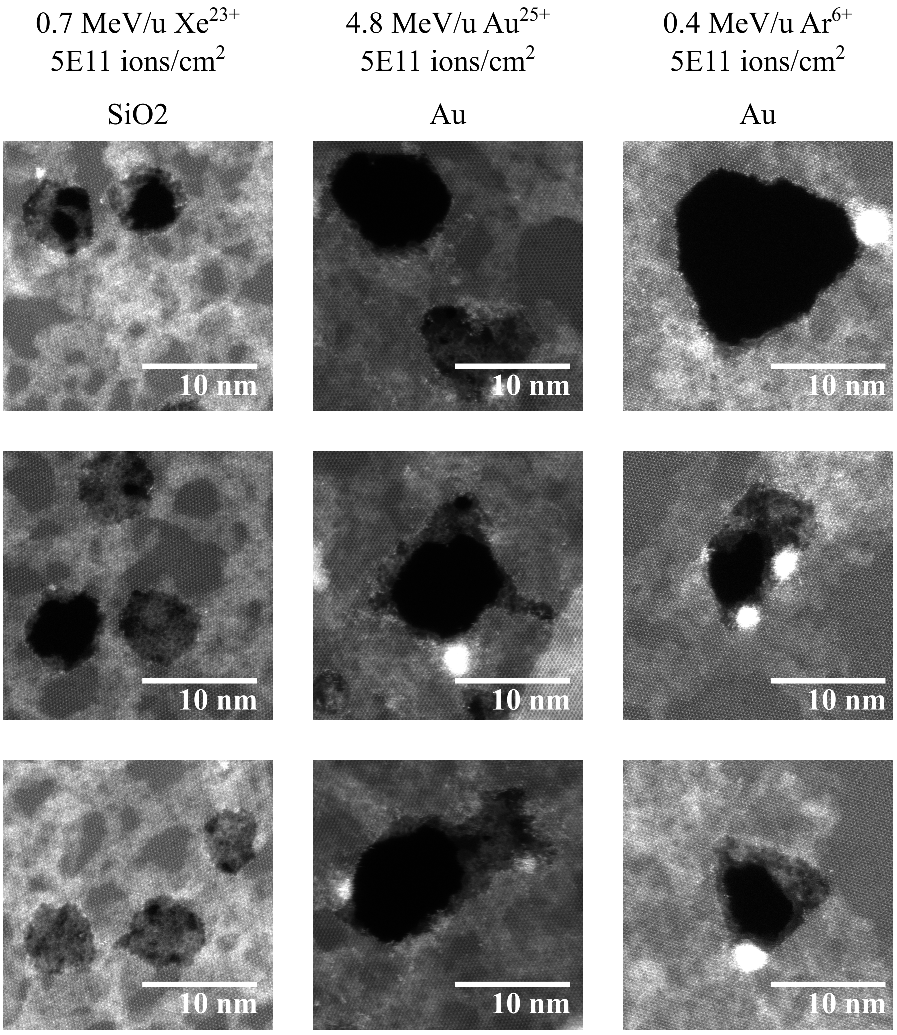

Figure S6: Representative STEM ADF images of pores formed in MoS_2 irradiated on SiO_2 and Au. Pores on Au show a broader range of shapes and more frequent lattice-aligned edges than pores on SiO_2, consistent with modification during Au removal and transfer.

Pore size distribution on different substrates

As shown in Fig. 4 in the main manuscript, while the SHI irradiation of MoS_2 on SiO_2 and Au creates the same mean pore radii, their standard deviation varies strongly. This is further analyzed by comparing the pore radius histograms of two samples. The histograms are shown in Fig. S7 and illustrate the much broader distribution on Au. Here, irradiation on SiO_2 creates highly uniform pores (R_{mean} = 3.29 ± 0.39 nm), while the distribution on Au is considerably wider (R_{mean} = 3.25 ± 1.15 nm). The histogram of the 0.4 MeV/u Ar^{6+} is not shown here, as the small efficiency

reduced the overall pore count considerably (to around 50 pores). Nevertheless, whithin this smaller dataset, a similar standard variation in pore size as in the 4.8 MeV/u Au^{25+} irradiation was found.

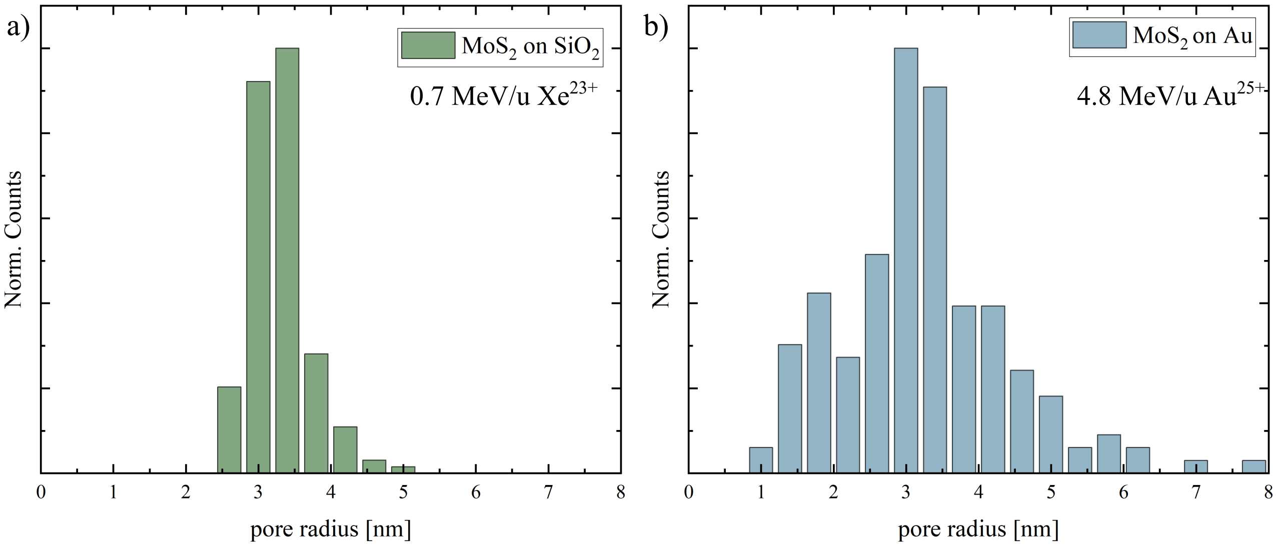

Figure S7: Histograms of pore radii for SHI-irradiated MoS_2 on SiO_2 (a) and Au (b). Although the mean pore radii are similar, the distribution for MoS_2 on Au is substantially broader, consistent with the greater variability in pore morphology after transfer from Au. Pore count in both histograms is ~150 pores.
